# Human herpes viruses are associated with steeper age-dependent increases of serum biomarkers for dementia in cognitively unimpaired women

**DOI:** 10.1038/s41598-025-10102-1

**Published:** 2025-07-15

**Authors:** Lisa M. James, George Stratigopoulos, Apostolos P. Georgopoulos

**Affiliations:** 1 The Healthy Brain Aging Group, Brain Sciences Center, Department of Veterans Affairs Health Care System, Minneapolis HCS, One Veterans Drive, Minneapolis, MN 55417 USA; 2 Department of Neuroscience, University of Minnesota Medical School, Minneapolis, MN USA; 3 Department of Psychiatry, University of Minnesota Medical School, Minneapolis, MN USA; 4 Institute for Health Informatics, University of Minnesota Medical School, Minneapolis, MN USA

**Keywords:** Blood biomarkers, Dementia, Human herpes viruses, Cognitively unimpaired aging, Biomarkers, Viral infection, Dementia

## Abstract

Blood biomarkers for dementia are being increasingly used for screening and possibly early detection of dementia in cognitively unimpaired (CU) people. Here we measured blood serum levels of 5 dementia-related biomarkers (Aβ_1–40_ [Aβ40], Aβ_1–42_ [Aβ42], Aβ42/Aβ40 ratio, phosphorylated Tau181 [pTau181], and phosphorylated Tau217 [pTau217]) and determined the seroprevalence of 6 HHV (HHV1, HHV2, HHV3, HHV4, HHV5, HHV6) in 345 samples drawn at successive visits from 167 CU women 26–98 years old. All biomarkers except for Aβ42/Aβ40 increased significantly with age, particularly in those who were HHV seropositive. With respect to the biomarkers, the increase was highest for Aβ40 > Aβ42 > pTau217 > pTau181, and, with respect to HHV, the increase was highest for HHV4 > HHV6 > HHV1 > HHV2 > HHV5 (HHV3 was seropositive in all samples). Overall, the average normalized rate of increase of biomarkers with age was 2.15 × higher in the HHV seropositive vs. seronegative groups (P = 0.003, paired samples t-test). The presence of apolipoprotein E4 (apoE4) genotype did not have a significant effect on those rates. These findings document a link between prior viral infection and dementia-related blood biomarkers, adding support to the HHV hypothesis in developing dementia, irrespective of apoE4 allele presence.

## Introduction

The factors that initiate sporadic Alzheimer’s disease, the most common form of late-onset dementia, remain unknown. While the apoE4 allele is a well-established risk factor^1–3^ and lifestyle influences risk^4,5^, it is widely believed that certain insults or “hits” may trigger or contribute to disease development, including exposure to various microbes^6^. Among these, human herpesviruses (HHVs) have been implicated^7,8^, particularly HHV1^9–11^, HHV3^9,11–14^, HHV4^15^, HHV6^15–17^, and HHV7^14,17^. The HHV-dementia hypothesis is supported by postmortem detection of HHVs in the brains of Alzheimer’s patients^8^, immunogenetic epidemiological studies^14,16^, and evidence that antiviral therapy may help prevent dementia onset^9^.

In recent years, technological advancements have driven substantial progress in research on blood biomarkers of dementia^18,19^. The most frequently studied biomarkers include amyloid-beta (Aβ40, Aβ42) and phosphorylated tau (pTau181, pTau217) proteins, which have been shown to correlate with positron emission tomography (PET) and cerebrospinal fluid (CSF) levels^20,21^. These biomarkers exhibit superior diagnostic accuracy compared to standard primary care evaluations^22^ and offer a less invasive and more cost-effective alternative to PET imaging and CSF collection. Furthermore, emerging evidence suggests that biomarker changes can precede clinical symptoms and dementia diagnosis by up to 20 years^23,24^, highlighting their prognostic potential.

To further explore the relationship between HHV infection and dementia risk, we investigated whether age-dependent changes in dementia blood biomarkers are influenced by prior HHV infection, as assessed by the presence of HHV-specific IgG antibodies in cognitively unimpaired (CU) individuals. Specifically, we evaluated five dementia blood biomarkers—Aβ40, Aβ42, Aβ42/Aβ40 ratio, pTau181, and pTau217—in 345 serum samples from CU women aged 26–98 years old. This study aims to provide new insights into the potential interplay between HHV exposure and neurodegenerative processes, offering a novel perspective on the early detection and risk assessment of dementia.

## Results

### Age

**Fig. 1:**
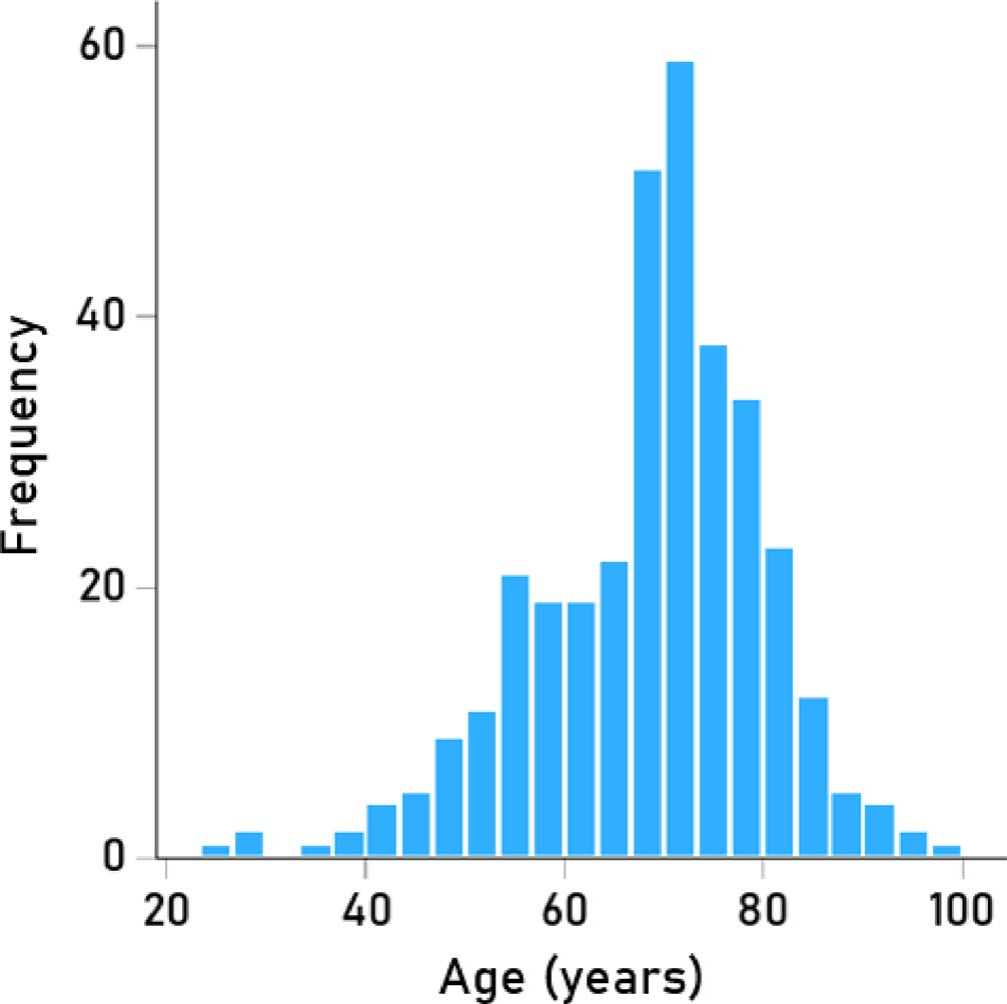
Frequency distribution of age at visit. N = 345.

The frequency distribution of age of the participants at each visit is shown in Fig. 1 (mean ± SD: 68.6 ± 11.6 y, range 25.7–95.8 y, N = 345).

### MoCA

MoCA scores did not differ significantly between the apoE [22/23] group (mean ± SEM 27.75 ± 0.237, N = 44) and the apoE [44/34] (27.67 ± 0.164, N = 87) group (*P* = 0.771, independent sample t-test). However, MoCA scores significantly declined with age (Spearman rho = − 0.242, *P* < 0.0001). This age-related decline was 1.8 × steeper in the apoE [44/34] group, as indicated by the linear regression slope of MoCA scores against age: − 0.052 MoCA score/y for apoE [44/34] (*P* < 0.001, N = 87) vs. − 0.044 for apoE [22/23] (*P* = 0.056, N = 44).

### Biomarkers

**Fig. 2:**
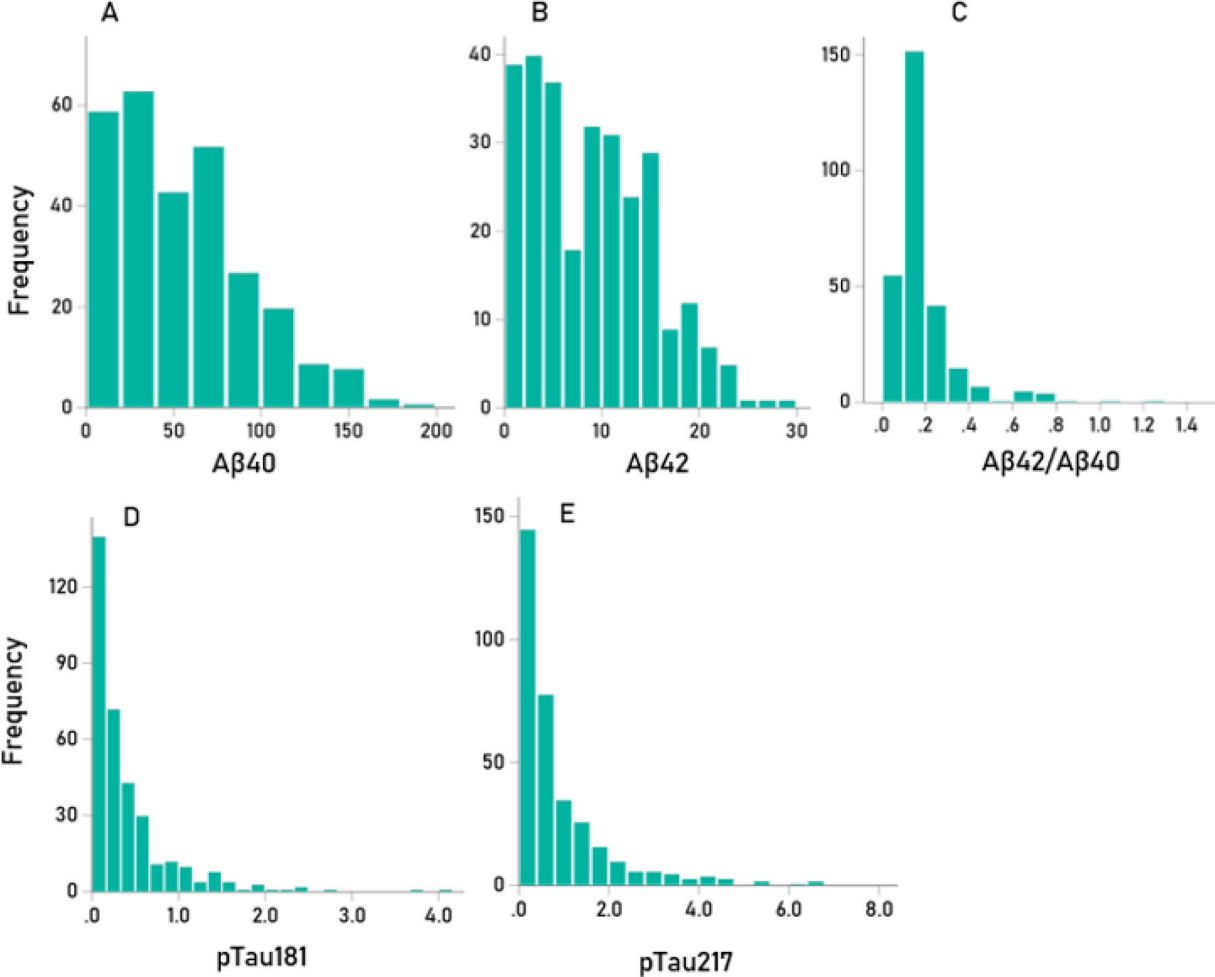
Frequency distributions of the 5 biomarkers used. (**A**) Αβ40 (N = 323) (**B**) Αβ42 (N = 323); (**C**) Αβ42/Αβ40 (N = 311); (**D**) pTau181 (N = 345); (**E**) pTau217 (N = 345).

**Fig. 3:**
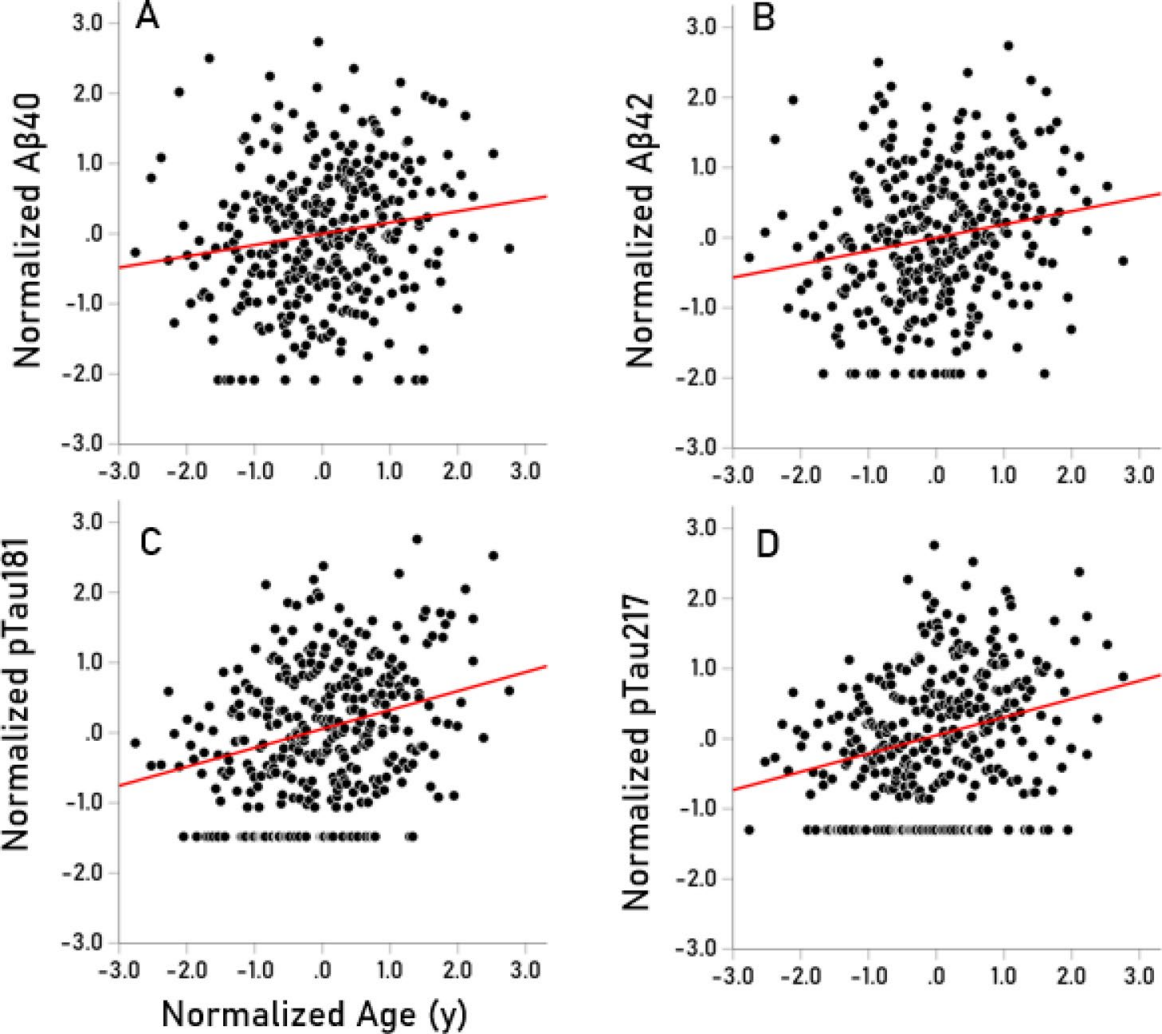
**(A**–**D)** Scatter plots of normalized (van der Waerden normal score) biomarker values against age for the biomarkers indicated; their correlations are given in Table 3. Αβ42/Αβ40 did not change significantly with age and is not shown.

The frequency distributions of the 5 biomarkers used were skewed to the right, as illustrated in Fig. 2. Consequently, rank-based nonparametric statistics were applied for their analysis (Table 1). Our findings were as follows: (a) Αβ40, Αβ42, pTau181 and pTau217 were positively and significantly correlated, with the strongest associations observed between Αβ40 and Αβ42 (rho = 0.766) and pTau181-pTau217 (rho = 0.484); additionally, Aβ42/Αβ40 was significantly correlated with pTau217 (Table 2). (b) All biomarkers except Αβ42/Αβ40 (data not shown) increased significantly with age (Fig. 3); the strongest correlations were observed for pTau181 and pTau217 (Table 3). (c) The positive biomarker correlations remained significant after controlling for age (Table 4). (d) Biomarker levels did not differ significantly between the apoE[22/23] and apoE[44/34] groups (Table 5). Finally, (e) none of the biomarkers had a significant effect on MoCA scores (Table 6).

**Table 1:** Nonparametric descriptive statistics for the biomarkers used. IQR, interquartile range. Values for Aβ42/Αβ40 are pure numbers. All other values are pg/ml.

Measure	Median	IQR	N
Aβ40	51.770	56.020	287
Aβ42	8.631	9.620	287
Aβ42/Αβ40	0.149	0.104	287
pTau181	0.230	0.445	345
pTau217	0.524	1.106	345

**Table 2:** Spearman correlations (rho) among the 5 blood biomarkers studied. Since Aβ42/Αβ40 was derived from Aβ40 and Αβ42, no correlations are shown with the latter 2 biomarkers.

		Aβ42	pTau181	pTau217
Αβ40	Rho	0.766	0.265	0.281
	*P* value	< 0.001	< 0.001	< 0.001
	N	323	323	323
Aβ42	Rho		0.265	0.281
	*P* value		< 0.001	< 0.001
	N		323	323
Aβ42/Αβ40	Rho		0.099	0.174
	*P* value		0.081	0.002
	N		311	311
pTau181	Rho			0.484
	*P* value			< 0.001
	N			345

**Table 3:** Spearman correlations (rho) of blood biomarkers with age (years).

	Aβ40	Aβ42	Aβ42/Αβ40	pTau181	pTau217
Rho	0.183	0.210	0.025	0.255	0.276
*P* value	0.001	< 0.001	0.660	< 0.001	< 0.001
N	323	323	311	345	345

**Table 4:** Rank-based partial correlations (RPC) among blood biomarkers, controlling for age.

		Aβ42	Aβ42/Αβ40	pTau181	pTau217
Αβ40	Rho	0.757	− 0.108	0.229	0.242
	*P* value	< 0.001	0.057	< 0.001	< 0.001
	N	323	311	323	323
Aβ42	Rho		0.478	0.265	0.281
	*P* value		< 0.001	< 0.001	< 0.001
	N		311	323	323
Aβ42/Αβ40	Rho			0.098	0.177
	*P* value			0.085	0.002
	N			311	311
pTau181	Rho				0.484
	*P* value				< 0.001
	N				345

**Table 5:** Lack of significant effect of apoE[22/23] vs. apoE[44,34] genotypes on blood biomarkers (ANCOVA with ranked biomarker level as the dependent variable, apoE2_4 as a fixed factor, and ranked age as covariate).

		*P* value
Aβ40	${\text{F}}_{[\text{1,123}]}=0.378$	0.540
Aβ42	${\text{F}}_{[\text{1,123}]}=0.548$	0.461
Aβ42/Αβ40	${\text{F}}_{[\text{1,116}]}=0.451$	0.503
pTau181	${\text{F}}_{[\text{1,128}]}=0.992$	0.321
pTau217	${\text{F}}_{[\text{1,128}]}=0.792$	0.375

**Table 6:** Lack of significant effect of blood biomarkers on MoCA, controlling for age. The values are RPC (rank-based partial correlations).

		MoCA
Aβ40	RPC	− 0.006
	*P* value	0.912
	N	320
Αβ42	RPC	− 0.05
	*P* value	0.374
	N	320
Aβ42/Αβ40	RPC	0.024
	*P* value	0.676
	N	311
pTau181	RPC	0.029
	*P* value	0.597
	N	342
PTau217	RPC	0.004
	*P* value	0.938
	N	342

### HHV seropositivity and its effect on the age-related increase in serum biomarkers

**Fig. 4:**
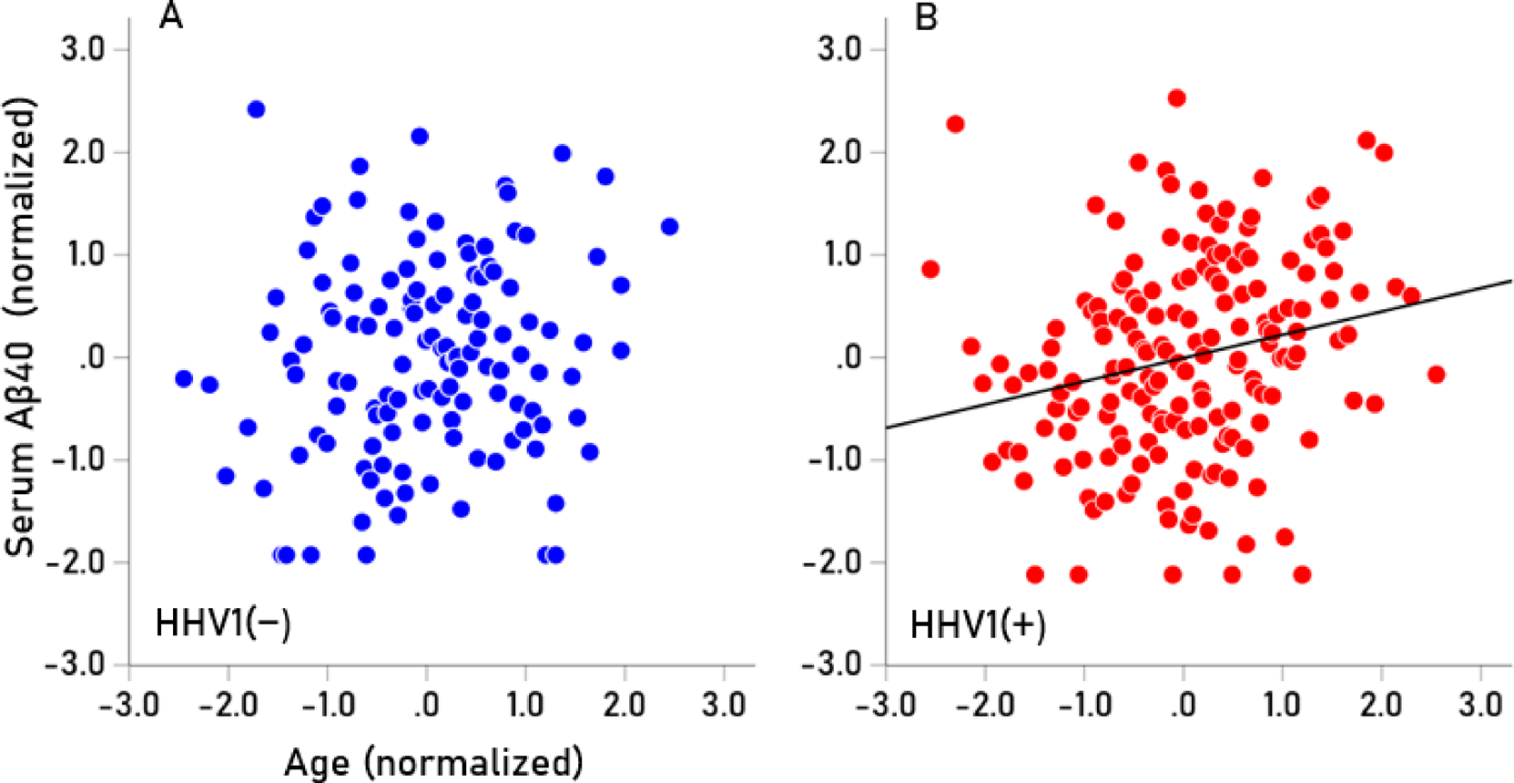
(**A**,**B**) Serum values of Αβ40 are plotted against age for HHV1(−) (N = 125; rho = 0.130) and HHV1(+) (N = 174; rho = 0.265), respectively.

**Fig. 5:**
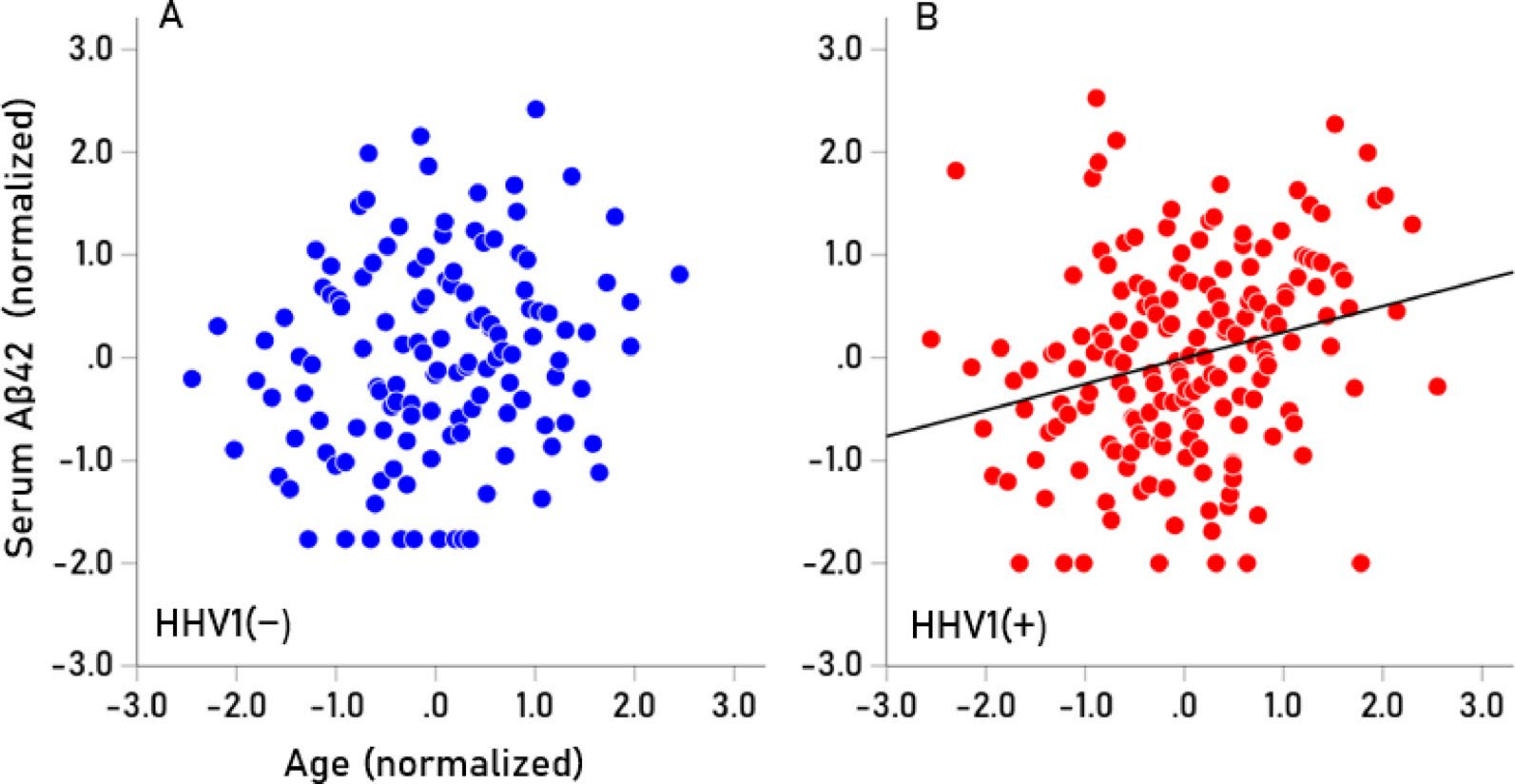
(**A**,**B**) Serum values of Αβ42 are plotted against age for HHV1(−) (N = 125; rho = 0.172) and HHV1(+) (N = 174; rho = 0.279), respectively.

**Fig. 6:**
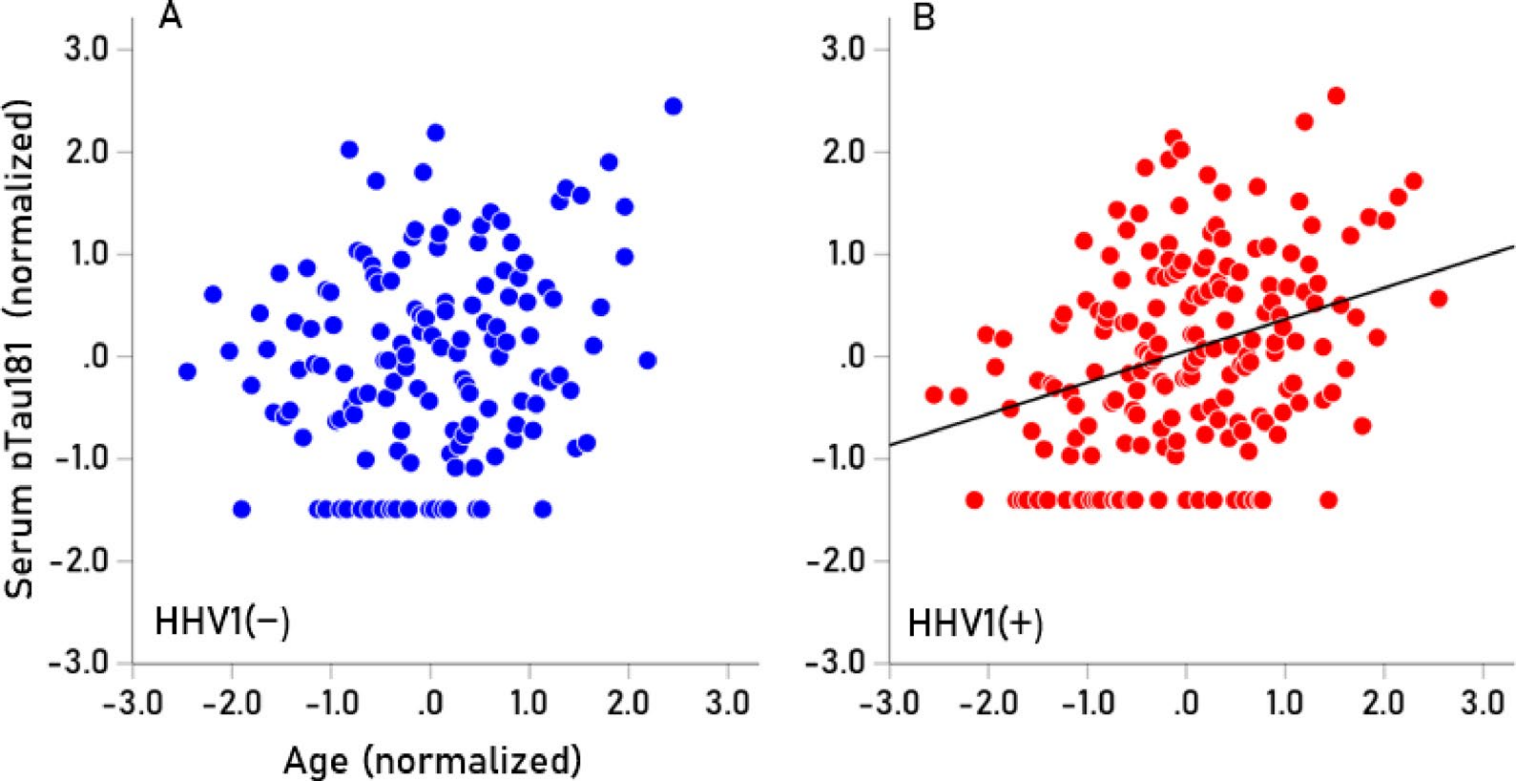
(**A**,**B**) Serum values of pTau181 are plotted against age for HHV1(−) (N = 125; rho = 0.176) and HHV1(+) (N = 174; rho = 0.306), respectively.

**Fig. 7:**
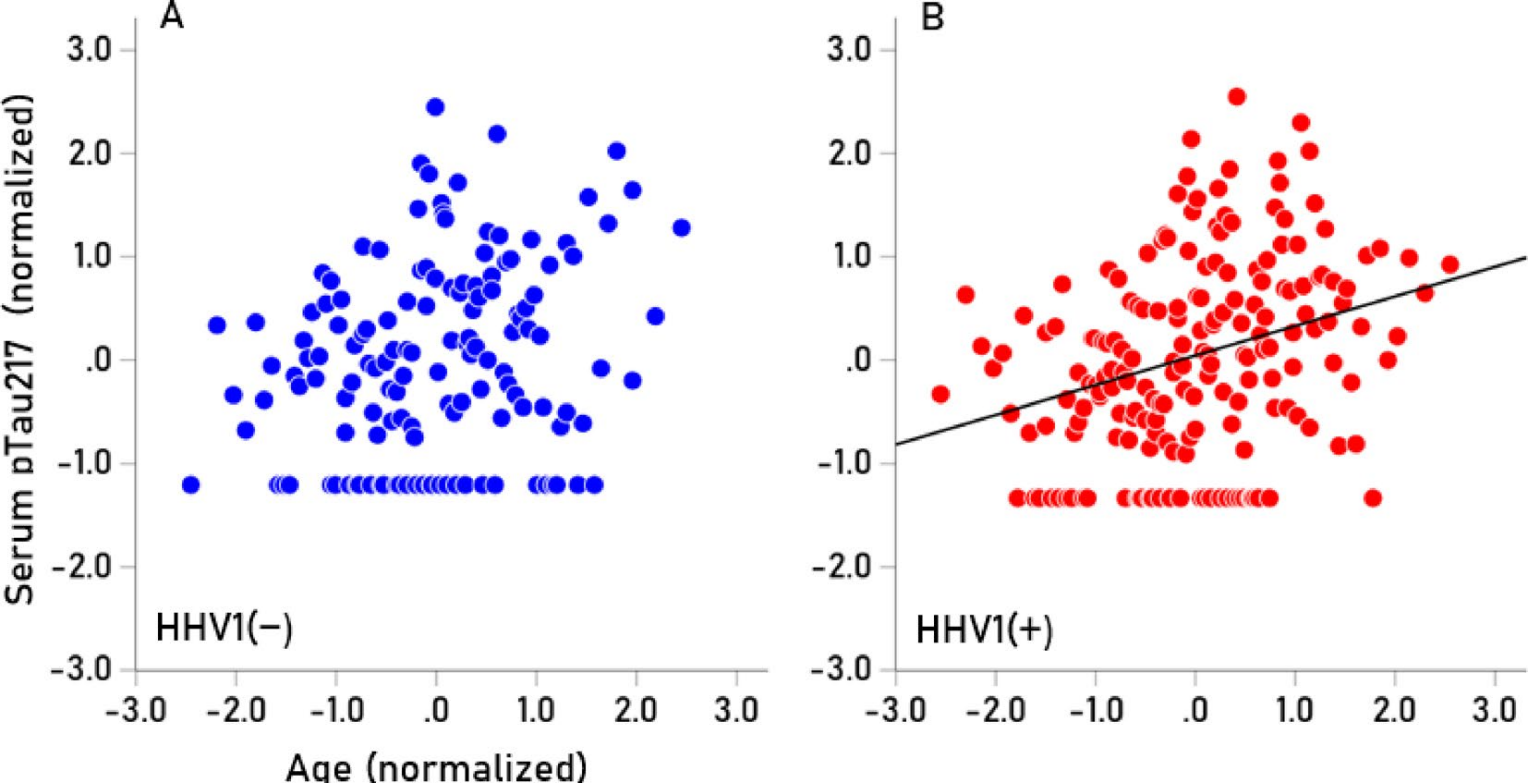
(**A**,**B**) Serum values of pTau217 are plotted against age for HHV1(−) (N = 125; rho = 0.228) and HHV1(+) (N = 174; rho = 0.333), respectively.

Seropositivity rates varied among the HHV tested (Table 7). HHV3 had a 100% seropositivity rate, so no further analyses were conducted for this virus. For the remaining viruses (HHV1, HHV2, HHV4, HHV5, HHV6), we investigated whether virus seropositivity influenced the age-related increase we observed in the four serum biomarkers (Aβ40, Αβ42, pTau181, pTau217). Exploratory analyses indicated a greater age-related increase in biomarker levels in the HHV seropositive groups, an effect that we quantified as follows. First, a univariate analysis of covariance (ANCOVA) was performed for each biomarker, where Virus seropositivity (0, 1) was a fixed factor (main effect) and Virus x Age was an interaction term. We found the following. (a) The HHV × Age interaction was not significant for Aβ42/Aβ40, so no further testing was conducted for this biomarker. The interaction term was statistically significant in all other cases (*P* < 0.01, F-test in ANCOVA), indicating non-parallel slopes in biomarker vs. age regression. Therefore, Spearman’s rho correlations were calculated separately for seropositive and seronegative groups (Table 8). (b) In 85% of cases (17/20), correlations were higher in the seropositive group, a statistically highly significant preponderance (*P* < 0.001, Wald test for single sample proportion). Additionally, rho distributions differed significantly between seropositive and seronegative groups (*P* = 0.003, Wilcoxon signed-rank test). (c) Spearman’s rho can be interpreted as the slope in a linear regression of ranked biomarker values against age. The mean (± SEM) Spearman’s rho in the HHV(+) group was 0.266 ± 0.016, whereas in the HHV(−) group, it was 0.124 ± 0.041. This indicates that the average rate of biomarker increase with age was more than twice as high in the seropositive group (2.15 × higher, *P* = 0.003, paired t-test), confirming findings from the nonparametric Wilcoxon test. The normalized serum biomarker changes with age for HHV1(−) and HHV1(+) are illustrated in Figs. 4, 5, 6 and 7.

**Table 7:** Prevalence of seropositivity for the 6 HHVs investigated. Numbers in the column Total indicate the number of tests that gave unambiguous results in the ELISA.

HHV strain		N seropositive	Total	Percent seropositive
HHV-1	HSV1	185	324	57.1
HHV-2	HSV2	76	339	22.4
HHV-3	VZV	341	341	100.0
HHV-4	EBV	329	343	95.9
HHV-5	CMV	187	344	54.4
HHV-6	6A, B	300	329	95.4

**Table 8:** Spearman correlations (rho) of Aβ40, Αβ42, pTau181, pTau217 with age for the seronegative (−) and seropositive (+) virus groups.

		Seroprevalence	
Virus	Biomarker	Negative (−)	Positive (+)
HHV1	AB40	0.130	0.265
	AB42	0.172	0.279
	pTau181	0.176	0.306
	pTau217	0.228	0.333
HHV2	AB40	0.183	0.244
	AB42	0.188	0.337
	pTau181	0.289	0.146
	pTau217	0.206	0.492
HHV4	AB40	− 0.459	0.209
	AB42	0.014	0.216
	pTau181	0.385	0.236
	pTau217	− 0.031	0.282
HHV5	AB40	0.115	0.236
	AB42	0.161	0.246
	pTau181	0.235	0.262
	pTau217	0.268	0.267
HHV6	AB40	0.098	0.184
	AB42	− 0.178	0.247
	pTau181	0.053	0.269
	pTau217	0.244	0.271

**Fig. 8:**
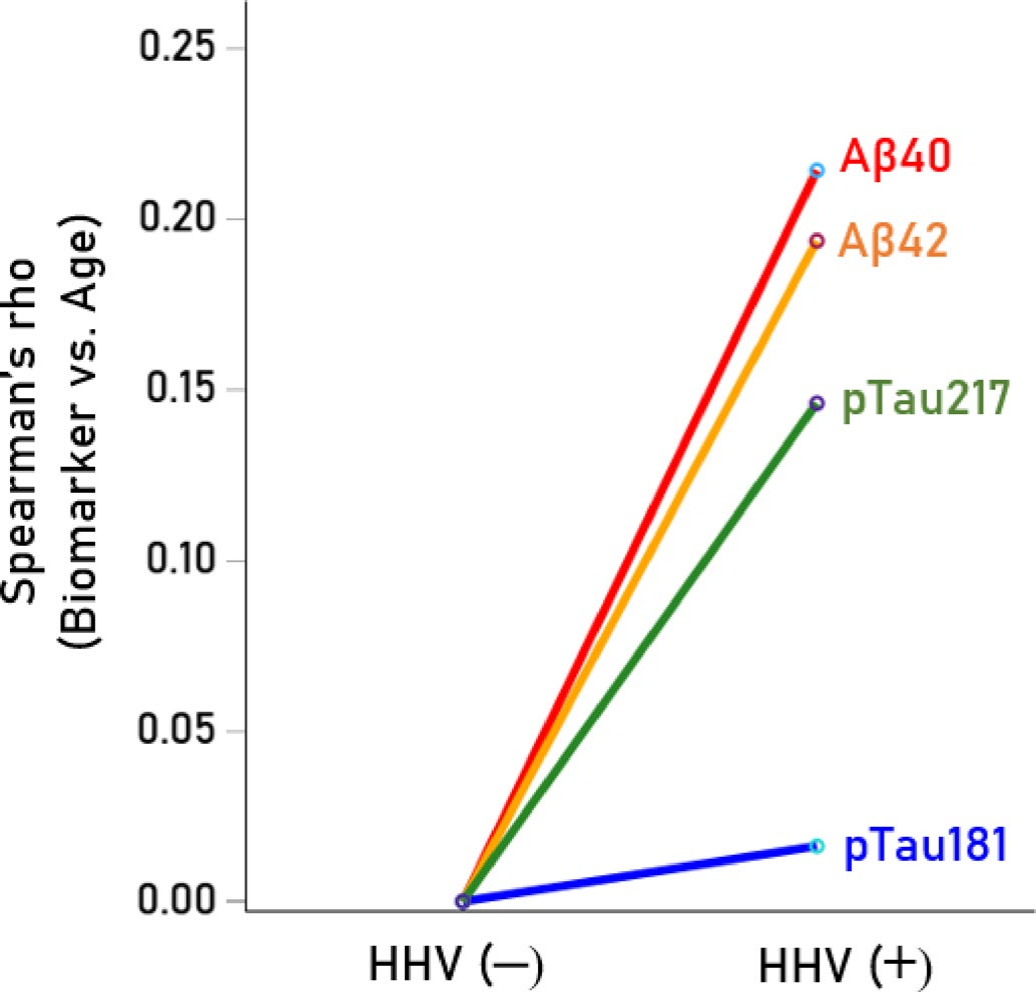
Average (across HHV) increase in Spearman correlation of biomarker vs. age in HHV(+) over HHV(−) for the 4 biomarkers shown. See text for details.

**Fig. 9:**
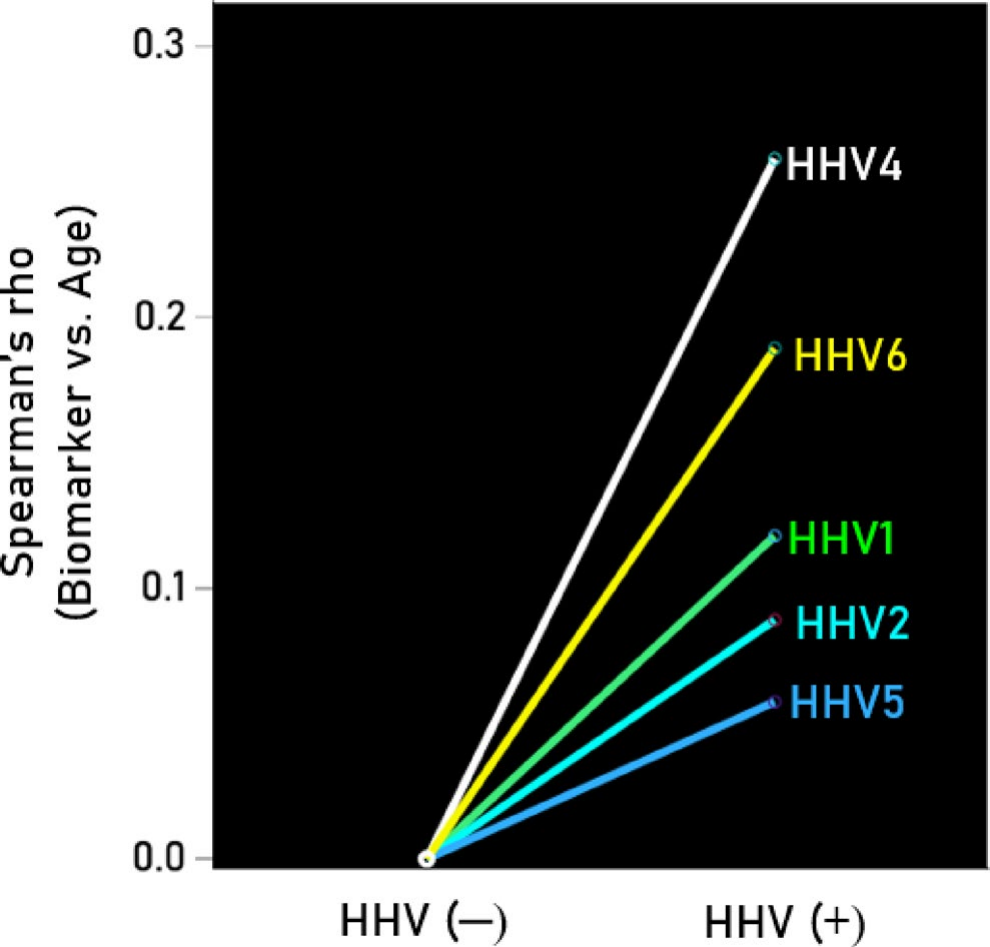
Average (across biomarkers) increase in Spearman correlation of biomarker vs. age in HHV(+) over HHV(−) for the 5 HHVs shown. See text for details.

With respect to biomarkers (across all HHVs), the greatest effect of viral seropositivity (i.e., the largest increase in rho: rho[HHV(+) − rho(HHV(−)] was seen for Aβ40 and the lowest for pTau181 (Aβ40 > Αb42 > pTau217 > pTau181); the average increases in rho for specific biomarkers are shown in Fig. 8. Finally, with respect to HHVs (across all biomarkers), the greatest effect of viral seropositivity (assessed as above) was observed for HHV4 and the weakest for HHV5 (HHV4 > HHV6 > HHV1 > HHV2 > HHV5); the average increases in rho for specific HHVs are shown in Fig. 9.

### HHV and apoE

The frequency of the 6 apoE genotypes in our participants is shown in Table 9. To test for an effect of the apoE4 vs. apoE2 alleles (predisposing and protective for Alzheimer’s disease, respectively) on the levels of the various biomarkers, we created two groups (Table 9), one comprising participants carrying isoforms carrying the apoE4 allele (44, 34; apoE[44/34], N = 87) and another comprising participants carrying the apoE2 allele (22, 23; apoE[22/23], N = 44); participants who did not carry apoE4 or apoE2 alleles (i.e. of the apoE33 genotype) were excluded as well as those with the apoE24 genotype, given the known opposite effects of the apoE4 and 2 alleles.

**Table 9:** ApoE genetic makeup of participants and apoE2, apoE4 groups.

ApoE alleles	Frequency		
22	2	apoE[22/23]	44
23	42		
24	4		
33	209		
34	73	apoE[44/34]	87
44	14		

For each HHV strain, we evaluated the potential association between the HHV prevalence and presence of apoE[22,23]/apoE[44,34] alleles by analyzing two-way (2 × 2) tables where the rows represented HHV(−)/HHV(+) counts, and the columns apoE[22,23]/apoE[44,34] counts. No statistically significant associations were found for any HHV strains (*P* > 0.05, Fisher’s exact test).

## Discussion

Here we measured blood serum levels of dementia biomarkers in cognitively unimpaired women and evaluated the influence of HHV seropositivity on these biomarkers. We found that all biomarkers increased with age except Αβ42/Αβ40, regardless of apoE genotype, and that HHV seropositivity was associated with a significantly steeper age-related increase in these biomarkers compared to those without prior infection. These findings contribute to the growing body of literature documenting the presence of blood biomarkers of dementia in individuals without cognitive impairment and highlight the potential role of common viral infections in modulating the levels of dementia-related biomarkers.

The development of ultra-sensitive techniques to detect blood biomarkers of dementia permits detection of biomarkers at low concentrations long before clinical symptoms emerge^25^. Given their prognostic value^19^, the ease of assessment compared to invasive methods, and validation against gold-standard PET or CSF^21,26–28^, emerging research is aimed at assessing dementia blood biomarkers in cognitively unimpaired individuals to identify those at pre-clinical stages who many benefit from monitoring and potentially early intervention. Although in its infancy relative to studies in clinical populations^29^, studies of blood biomarkers of dementia in cognitively unimpaired individuals have documented that plasma blood biomarkers predict cognitive decline, conversion to AD, brain volume loss and glucose hypometabolism^24,30–33^. In agreement with evidence we present here, prior research has shown that the influence of these biomarkers on subsequent cognitive decline is independent of apoE4 status^30,31,34^, and that apoE4 does not improve discriminatory accuracy for late-life dementia beyond mid-life dementia blood biomarkers^24^. These reports add to the utility of blood biomarkers in early identification of dementia risk. Objective indicators of risk for dementia are particularly important given that a substantial number of patients with cognitive impairment are not accurately identified in primary care settings, where the vast majority of dementia patients are managed, thereby limiting opportunities for early intervention^35^. While use of blood biomarkers of dementia in memory clinics has been recommended for several years^19^, their routine use in other settings is increasingly recognized as beneficial in terms of early screening, enhancing diagnoses, and informing treatment decisions^36,37^.

In this sample of cognitively unimpaired women, we found that all blood biomarkers of dementia tested increased with age, except for Αβ42/Αβ40. Previous studies have reported that plasma Aβ40 levels increase with age in healthy individuals^38^, and plasma Aβ42 levels also increase significantly with age in cognitively unimpaired individuals^39,40^. Other evidence suggests that in cognitively unimpaired individuals, plasma Aβ levels progressively increase with age, whereas in individuals who eventually develop dementia, Aβ levels are initially elevated in the pre-dementia stage but subsequently decline before symptom onset^38^. Prior research has also demonstrated an increase in pTau181 from midlife to late life, independent of cognitive status^24^, while other studies have shown that plasma pTau217 levels rise with age in cognitively unimpaired individuals who are Aβ+ but not in those who are Aβ−^41,42^. Since research evaluating blood biomarker changes with age in cognitively unimpaired individuals is in its infancy, additional research is warranted in order to characterize associations of blood biomarkers of dementia across the lifespan and to evaluate potential modulating factors.

Given the substantial evidence linking viral infections to dementia^6–17^, we evaluated the impact of HHV seropositivity on age-related changes in blood biomarkers of dementia in this cognitively unimpaired cohort. Our findings indicate that HHV seropositivity was associated with double the rate of biomarker increase with age, suggesting that HHV infection hastens processes linked to dementia. The greatest HHV-related increase was seen for Aβ40, followed by Aβ42, pTau217, and pTau181.

The significance of the HHV-associated increase in Aβ remains unclear. Traditionally, amyloid deposition has been considered an early event that triggers a cascade of neurobiological changes leading to cognitive decline^43^. Given that blood Aβ levels moderately correspond to CNS Aβ levels^44^, our findings raise concerns about an elevated risk of future dementia in HHV-seropositive individuals. However, that concern is somewhat mitigated by evidence documenting the presence of Aβ plaques in cognitively unimpaired normal adults^45,46^, that plasma Aβ42 is significantly decreased in individuals with mild cognitive impairment and Alzheimer’s disease patients compared to age-matched controls^38^, and that tau pathology is more strongly associated with cognitive decline than Aβ^19^. Conversely, mounting evidence indicates that Aβ may serve an antimicrobial function^47–49^. From this perspective, the observed increase in Aβ in our cognitively unimpaired sample—particularly among HHV-seropositive individuals—could reflect a protective response against infection. As reviewed elsewhere^50^, studies have demonstrated co-localization of Aβ plaques with viral DNA in brain tissues, shown that infection with HHV stimulates Aβ production, and found that overexpression of Aβ confers increased resistance to infection. Additionally, attempts to deplete Aβ have frequently resulted in adverse outcomes, including heightened susceptibility to infections. In this context, the age-related increase in Aβ, especially in the HHV-seropositive group, may reflect protective activity against HHVs that persists until Aβ accumulation exceeds the brain’s clearance capacity, potentially shifting from a protective to a pathological role^51,52^.

We also found that HHV seropositivity was associated with increased pTau, particularly pTau217. Blood pTau is strongly correlated with CSF pTau levels^53^, and blood pTau217 is considered a reliable marker of amyloid pathology^54^ although amyloid-independent increases in blood pTau have also been documented^55,56^. Furthermore, pTau217 has been linked to future cognitive decline in Aβ+ cognitively unimpaired individuals making it a key predictor of progression in the earliest stages of Alzheimer’s disease^57^. Notably, previous research has shown that HHV1 (i.e., herpes simplex virus) seropositivity is associated with increased PET Aβ load in cognitively normal older adults, though it was not linked to pTau181 or other neurodegeneration markers^58^. In our study, pTau217 exhibited a stronger correlation with Aβs than pTau181, and virus seropositivity was associated with greater age-related increase in both Aβs and pTau217 compared to pTau181.

With regard to viral influence, HHV seropositivity moderated the age-related increase in blood biomarkers of dementia with the strongest effect observed for HHV4, followed by HHV6, HHV1, HHV2, and HHV5. This age-related increase in blood biomarkers associated with HHV seropositivity is consistent with extensive evidence implicating viruses in dementia pathogenesis, potentially as causal factors^7^, the bulk of which has focused on HHV1/HSV1^9^. A recent prospective study in non-demented adults found that HSV1 seropositivity, but not HHV5 (cytomegalovirus; CMV) seropositivity, doubled the risk of dementia over a 15-year follow-up period^59^; however, other viruses were not investigated. Interestingly, in our study, HHV5 had the weakest impact on the age-related biomarker increases (Fig. 6), consistent with prior findings. While our results confirm the association between HHV1/HSV1 seropositivity and elevated dementia-related biomarkers, they also extend to other HHVs, particularly HHV4 and HHV6. This is consistent with previous research identifying HHV4 and HHV6 as potential contributors to dementia^15–17,60,61^.

In summary, the findings in this study suggest that common human herpes viral infections are associated with steeper age-related increase in blood biomarkers of dementia in cognitively unimpaired women. That is, HHV infection hastens accumulation of dementia biomarkers. As such, HHV-infected individuals may surpass established AD biomarker cutoffs at an earlier age than uninfected individuals. Notably, these findings were in the absence of kidney disease, diabetes, and untreated hypertension, all of which have been associated with increased dementia biomarkers^62–65^. Despite the novelty of these findings, the study is not without limitations. For example, while this study focused on HHV infections, other infectious agents not investigated here may also influence dementia-related blood biomarkers. Indeed, various viral (e.g., influenza, flaviviruses, and coronaviruses) and bacterial infections have been linked to increased dementia risk^7,9,50^. In addition, the current study was limited to women in the Upper Midwest of the United States. In light of sex differences in HHV seroprevalence^66^, immune response^67^, and dementia prevalence^68^, it is unknown whether the current findings regarding the influence of HHV seroprevalence on dementia biomarkers generalize to men. Furthermore, global differences in HHV seroprevalence^69^ and dementia prevalence^70^ may similarly limit generalizability. Also, findings from the present study which focused on Aβ40, Aβ42, pTau181, and pTau217 may not extend to other biomarkers (e.g., pTau231, neurofilament light chain, or glial fibrillary acidic protein). Finally, the present study does not address the fact that HHVs, along with many other infectious agents, are nearly ubiquitous, yet not all infected individuals develop dementia. This discrepancy suggests that additional factors not investigated here likely modulate the relationship between HHV infection and dementia risk. Beyond the well-documented role of apoE, genetic variability in Human Leukocyte Antigen (HLA)—which plays a crucial role in immune response—may influence individual susceptibility to infection and the corresponding impact on dementia biomarkers. The investigation of these genetic and immunological factors represents an important direction for future research.

## Materials and methods

### Participants

A total of 167 cognitively healthy women participated in the study as paid volunteers. The data were obtained as part of an ongoing longitudinal study involving annual data acquisition; consequently, the number of annual visits varied for participants, for a total of 345 visits. Women were excluded from participation if they had been diagnosed at any point in their lifetime with a neurological disorder, any autoimmune disorder associated with neurocognitive dysfunction (e.g., systemic lupus erythematous, rheumatoid arthritis), any major medical condition affecting brain function (e.g., brain cancer, head injury with cognitive sequelae), serious psychiatric diagnoses (e.g., bipolar disorder, schizophrenia, any history of psychiatric hospitalization), or any recent/current medication or treatment known to affect brain functioning (e.g., radiation, chemotherapy). Written informed consent was obtained from study participants. The institutional review board and relevant committees of the Minneapolis VA Health Care System approved the study protocol which was performed in accordance with the Declaration of Helsinki.

### Cognitive assessment

The Montreal Cognitive Assessment (MoCA)^71^ was administered to participants to screen for cognitive impairment. The MoCA assesses several domains of cognitive function including executive function, memory, language, and abstract reasoning, among others. Scores for each domain are added to reflect a total score ranging from 0 to 30. At recruitment, all participants had a total MoCA score (without the education point) ≥ 25.

### ApoE genotyping

Determination of apoE genotype was performed as follows for 344/345 participants (apoE could not be determined in one participant due to technical reasons). DNA samples were genotyped using PCR amplification followed by restriction enzyme digestion^72^. Each amplification reaction contained PCR buffer with 15 mmol/L MgCl_2_ ng amounts of genomic DNA, 20 pmol apoE forward (5N TAA GCT TGG CAC GGC TGT CCA AGG A 3N) and reverse (5N ATA AAT ATA AAA TAT AAA TAA CAG AAT TCG CCC CGG CCT GGT ACA C 3N) primers, 1.25 mmol/L of each deoxynucleotide triphosphate, 10% dimethylsulfoxide, and 0.25 μL Amplitaq DNA polymerase. Reaction conditions in a thermocycler included an initial denaturing period of 3 min at 95 C, 1 min at 60 C, and 2 min at 72 C; followed by 32 cycles of 1 min at 95 C, 1 min at 60 C, and 2 min at 72 C; and a final extension of 1 min at 95 C, 1 min at 60 C, and 3 min at 72 C. PCR products were digested with *HhaI* and separated on a 4% Agarose gel which was stained with Ethidium Bromide. Known apoE isoform standards were included in the analysis. Known apoE isoform standards were included in the analysis.

### Determination of blood biomarkers in serum

We determined the serum levels of the following dementia-related biomarkers: Aβ40, Aβ42, pTau181, and pTau217 as follows. Aβ40, Aβ42 were measured using the FUJIFILM Wako ELISA kits (Catalog # 296-64401 and 298-64601). pTau181 and pTau217 were assessed on a MESO SECTOR S 600MM using the S-PLEX Human Tau (pT181) and (pT217) kits (Meso Scale Discovery, Catalog # K151AGMS and K151APFS). Assays were performed and values calculated following the manufacturers’ instructions. Serum samples were diluted 1:2 by the diluent provided by the manufacturer.

### HHV seropositivity assays

We determined the seroprevalence of IgG antibodies against Human Herpes Virus 1(HHV1), HHV2, HHV3, HHV4, HHV5 and HHV6 using commercially available Enzyme-Linked Immunosorbent Assay (ELISA) kits; we could not obtain reliable results from kits for HHV7 and HHV8. The ELISA tests were performed as per the manufacturer’s instructions and recommendations. Serum samples were processed using a mini-automated 5-in-1 workstation (Crocodile cat. 84024-01; Berthold Technologies, Oak Ridge, TN, USA). The workstation includes the ELISA microtiter plate reader which was read at dual wavelengths for absorbance at 450 nm and 620 nm as reference wavelengths. Details of the virus-specific ELISA kits are as follows. (a) HHV1: Human Anti-Herpes simplex virus Type 1 IgG ELISA Kit (HSV1), Abcam Inc., Boston, MA USA, cat. ab 108737; (b) HHV2: Human Anti-Herpes simplex virus Type 2 IgG ELISA Kit (HSV2) Abcam cat. ab 108739; (c) HHV3: Human Anti-Varicella-Zoster virus IgG ELISA Kit (VZV) Abcam cat. ab 108782; (d) HHV4: Human Anti-Epstein Barr virus IgG ELISA Kit (EBV-VCA) Abcam cat. ab 108730; (e) Human Anti-Cytomegalovirus IgG ELISA Kit (CMV) Abcam cat. ab 108724; (f) Human Herpesvirus 6 IgG ELISA Kit cat. KA1457, Abnova, Taiwan. Ambiguous readings were not used in the analyses.

### Statistical analyses

Standard statistical methods were used, including descriptive statistics, analysis of covariance (ANCOVA), linear regression, and nonparametric statistics, including Spearman (rho) correlation. All statistical analyses were performed on ranked values. For visualization purposes, data were normalized using the van der Waerden normal score transformation^73^. The IBM-SPSS statistical package (version 30.0.0.0 172) was used for all statistical analyses. All P-values reported are 2-sided, $a=0.05$.

## Data Availability

The data that support the findings of this study are available from the corresponding author upon reasonable request.
